# Role of warm ocean conditions and the MJO in the genesis and intensification of extremely severe cyclone Fani

**DOI:** 10.1038/s41598-021-82680-9

**Published:** 2021-02-11

**Authors:** Vineet Kumar Singh, M. K. Roxy, Medha Deshpande

**Affiliations:** 1 Indian Institute of Tropical Meteorology, Ministry of Earth Sciences, Pune, India; 2 Department of Atmospheric and Space Sciences, Savitribai Phule Pune University, Pune, India

**Keywords:** Natural hazards, Ocean sciences

## Abstract

Cyclone Fani, in April 2019, was the strongest pre-monsoon cyclone to form in the Bay of Bengal after 1994. It underwent rapid intensification and intensified quickly to an extremely severe cyclone. It maintained a wind speed of ≥ 51 m s^−1^ (≥ 100 knots) for a record time period of 36 h. The total lifespan of the cyclone was double than the climatological lifespan. Also, the duration of the cyclone in its extremely severe phase and the accumulated cyclone energy were significantly larger than the climatological records for the pre-monsoon season. In the current study, we investigate the ocean-atmospheric conditions that led to its genesis, rapid intensification and long lifespan. Our analysis shows that the Madden Julian Oscillation and anomalous high sea surface temperatures provided conducive dynamic and thermodynamic conditions for the genesis of cyclone Fani, despite forming very close to the equator where cyclogenesis is generally unlikely. Further, favourable ocean subsurface conditions and the presence of a warm core eddy in the region led to its rapid intensification to an extremely severe cyclone. A large area of warm ocean surface and subsurface temperatures aided the cyclone to maintain very high wind speed for a record time period. The vital role of the ocean surface and the subsurface in the genesis and the intensification highlights the need to efficiently incorporate ocean initial conditions (surface and sub-surface) and ocean–atmosphere coupling in the operational cyclone forecasting framework.

## Introduction

Tropical cyclones are among the most destructive natural disasters on earth. The north Indian Ocean, including the Arabian Sea and the Bay of Bengal, accounts for about 6% of the global tropical cyclones^1^. The cyclone frequency in this region varies between 1–3 in the pre-monsoon (March–May) to 2–5 cyclones in the post-monsoon (October–December) season^2^. Studies show that there is an increase in the intensity of pre-monsoon cyclones in the Bay of Bengal during recent decades^3^. Since 1990, four major cyclones with a maximum sustained wind speed larger than 51 m s^−1^ (100 knots) have formed in this basin during the pre-monsoon months (March–May) (Source: Regional Specialised Meteorological Centre reports http://www.rsmcnewdelhi.imd.gov.in). Cyclone Fani, which formed as a depression on 26th April 2019 in the south Bay of Bengal, is the fifth major cyclone after 1990, and the strongest pre-monsoon cyclone after 1994 in the Bay of Bengal. It formed very close to the equator, intensified rapidly and attained a maximum wind speed of 59 m s^−1^ (115 knots). On 3rd May, it hit Odisha Coast near Puri with a wind speed of ~ 54 m s^−1^ (105 knots). It is for the first time since 1990 that a cyclone which formed in April hit the Odisha coastline. Cyclone Fani sustained for 204 h, which is double the average lifespan (100 h) of a pre-monsoon cyclone in the Bay of Bengal. Also, during its lifespan, it travelled a distance of 3024 km from the south Bay of Bengal (close to the equator) to the north Odisha coast and then to Bangladesh after striking the Odisha coast. The extremely high wind speed combined with coastal storm surge and flooding caused vast damage to infrastructure, agriculture and an estimated death of 70–90 human beings.

The Madden–Julian Oscillation (MJO) is known to influence cyclogenesis across the tropics^4–10^. In the north Indian Ocean also, the genesis and evolution of the cyclone is generally associated with the MJO^11,12^. The MJO is an eastward propagating band of enhanced convection close to the equator, followed by suppressed convection in that region at a 30–60 days cycle^13^. It is generally associated with the oscillation between low level easterly and westerly winds close to the equator. Also, corresponding variations are observed in the outgoing longwave radiation (OLR), which is a proxy of convection, with anomalous low values of OLR denoting enhanced convection. Further, the enhanced convection due to the MJO activity is associated with the strengthening of westerly winds in the lower troposphere to the west of the convection centre^14^. This leads to an increase in the lower level cyclonic vorticity close to the equator in both hemispheres. The anomalous westerlies associated with the MJO leads to the growth of small scale slow-moving eddies through barotropic eddy kinetic energy conversion from the mean westerly flow. These eddies, along with enhanced cyclonic vorticity, high sea surface temperatures (SSTs), and strong low-level convergence, provide conducive conditions for the genesis of the cyclone^15^. The cyclones in the north Indian Ocean are found to get clustered in the area of enhanced low level cyclonic vorticity associated with the MJO^16^. Another study shows that the seed for the genesis of cyclone in the north Indian Ocean provided by the MJO is the convectively coupled Rossby wave that detaches from the central area of MJO-induced convection^17^. MJO activity in the Indian Ocean increases the relative humidity and decreases the vertical wind shear, thus providing conducive background conditions for the genesis of the cyclone^18,19^. The chances of cyclone formation over the Bay of Bengal get significantly enhanced when the MJO associated enhanced convection is centered over the eastern Indian Ocean and the Maritime continent^19^. These studies show that the MJO plays a significant role in the genesis of cyclones in the north Indian Ocean. The unprecedented nature of cyclone Fani has motivated us to explore the role of MJO and other ocean-atmospheric processes in terms of its genesis very close to equator, its intensity, rapid intensification, and high wind speed for record duration.

## Materials and methods

Cyclone data such as wind speed and track information are obtained from the India Meteorological Department (IMD) Regional Specialised Meteorological Centre (RSMC) preliminary report on Fani cyclone. To represent the combined strength and duration of the tropical cyclone, we use the accumulated cyclone energy (ACE) index. ACE is calculated by summing the squares of the 6-hourly maximum wind speed in knots for the duration when the system has a wind speed of 35 knots (18 m s^−1^) or higher. The number is divided by 10,000 to make it more readable and easy to interpret^20^. ACE is estimated as:

1 $${\text{ACE}} = {1}0^{{ - {4}}} \sum {\text{v}}^{{2}}$$

where v is the maximum wind speed in knots. The ACE and other cyclone-specific information (such as the lifespan of cyclones in the Bay of Bengal) used in this study are estimated based on the reports from IMD RSMC.

For analysing the ocean surface conditions over the north Indian Ocean during the evolution of cyclone Fani, daily SST data from the Optimum Interpolation Sea Surface Temperature (OISST) data set, at a spatial resolution of 0.25°^21^, is utilized. The wave height data is obtained from the Indian National Centre for Ocean Information Services. Daily data for sea level anomalies are obtained from the Copernicus Marine Service Altimeter satellite gridded Sea Level Anomalies L4 dataset for the period 1993–2019. To investigate the ocean subsurface conditions that led to the intensification of the cyclone, we estimate the Tropical Cyclone Heat Potential (TCHP). TCHP is the integrated heat content per unit area relative to the 26 °C isotherm^22,23^. It is calculated as.

2 $${\text{TCHP }} =\uprho {\text{Cp}}\int_{0}^{Z26} {\left( {T - 26} \right)dz}$$

where C_p_ (4178 J kg^−1^ °C^−1^) is the heat capacity of ocean water at constant pressure, ρ (1026 kg m^−3^) is the average ocean water density in the upper ocean, and Z_26_ is the depth of 26 °C isotherm in the ocean. In our study, TCHP is calculated based on the ocean subsurface daily data obtained from Indian National Centre for Ocean Information Services—Global Ocean Data Assimilation System (INCOIS-GODAS)^24^ for the period 19th–25th April 2019.

In order to explore the atmospheric conditions prevailing during the evolution of the cyclone, we used the relative vorticity and wind data (at 850 and 200 hPa levels), obtained from the ERA-Interim reanalysis dataset at a spatial resolution of 0.75°^25^. The OLR data is obtained from NOAA interpolated OLR dataset^26^. The vertical wind shear of horizontal winds is the magnitude of vector difference in the winds at 200 hPa and 850 hPa levels. The daily anomalies of all atmospheric parameters and SST are calculated based on the daily climatology for the period 1982–2019. Student’s two-tailed *t* test is applied to identify the significance of the ocean–atmosphere parameters anomalies at 95% confidence level.

To identify the phase and amplitude of MJO and its role on the genesis and evolution of the cyclone, Real-time Multivariate MJO (RMM) index data obtained from the Australian Bureau of Meteorology is used. The different phases of RMM indicate the region in which the convection associated with the MJO is present. The RMM index is derived using the first two empirical orthogonal functions (RMM1 and RMM2) of the equatorially averaged over the region 15°S–15°N for OLR data, and lower level (850 hPa) and upper level (200 hPa) zonal wind data^27^.

## Results

### Characteristics of cyclone Fani

**Figure 1:**
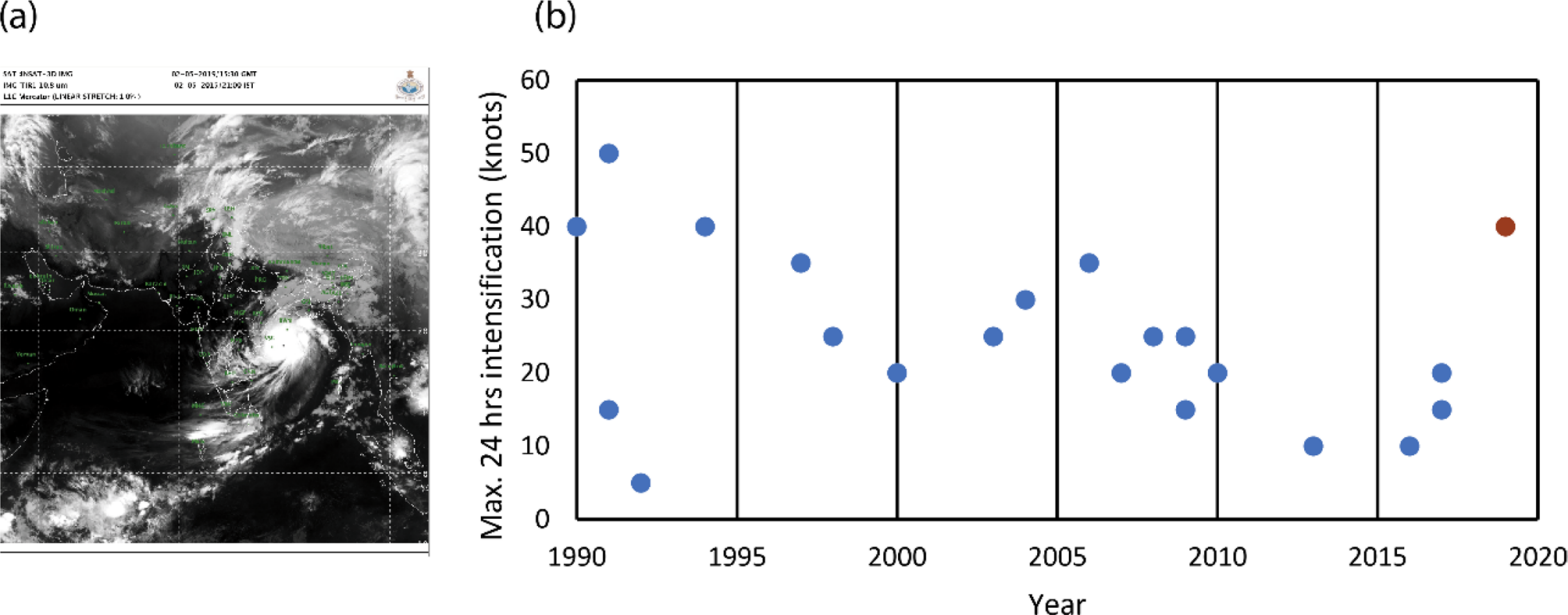
(**a**) INSAT-3D satellite image of cyclone Fani as on 2nd May 0900 p.m. IST (image obtained from India Meteorological Department, Ministry of Earth Science https://www.satellite.imd.gov.in/insat.htm) (**b**) Maximum 24 h intensification (in knots) by the pre-monsoon cyclones in the Bay of Bengal during their lifetime for the period 1990–2019. Cyclone Fani is shown by red circle.

**Figure 2:**
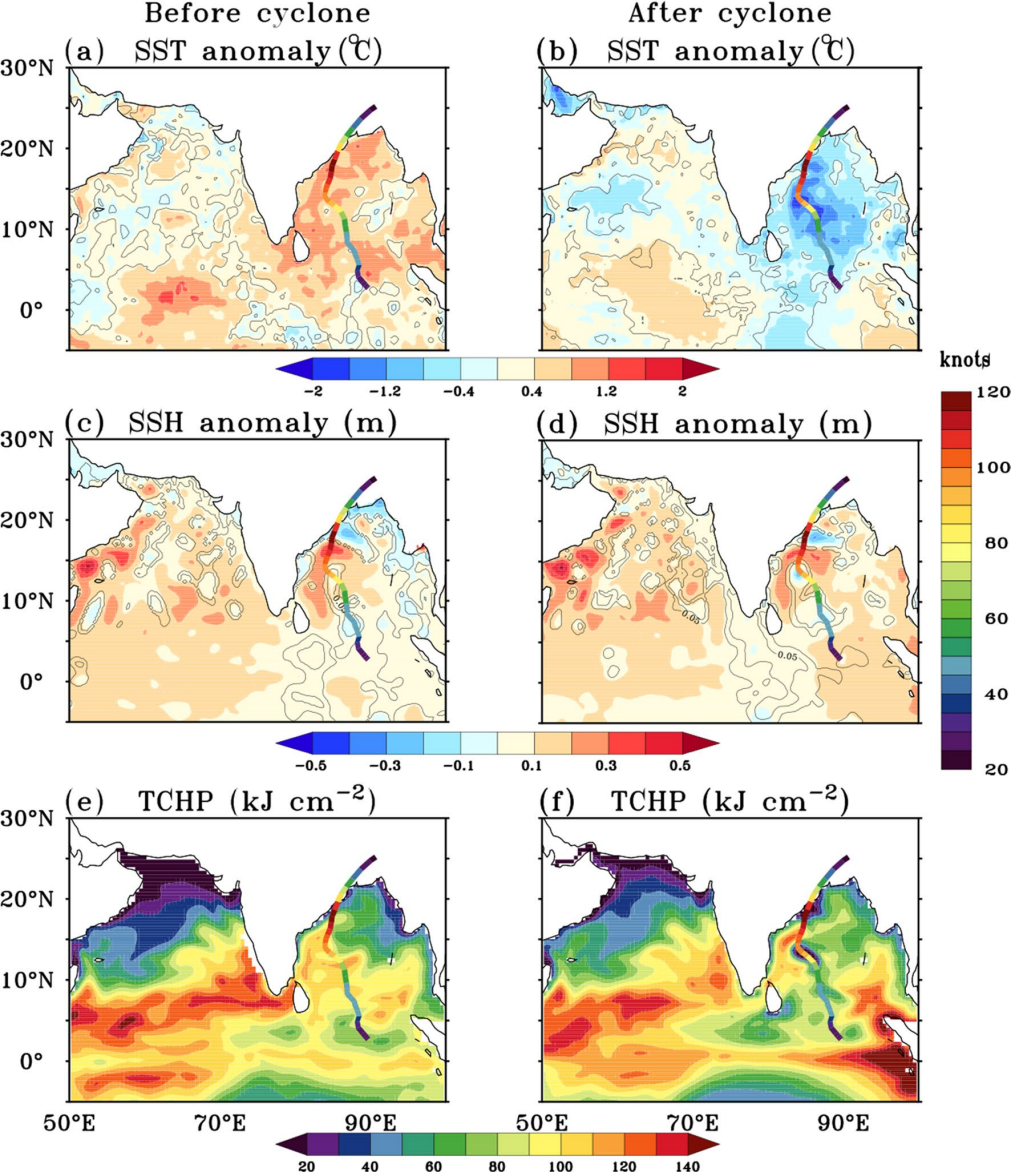
(**a**–**f**) SST (°C, shaded), SSH (m, shaded), TCHP (kJ cm^−2^, shaded) anomalies before and after cyclone Fani. Black contour lines in panels (**a**–**d**) denotes the 95% confidence level. The ocean parameters are averaged for the period 19 April–25 April 2019 and 4 May–10 May 2019 for preparing the anomalies before and after the cyclone. Colour along the track denotes the wind speed in knots, during each day of the cyclone. The figure is created using Ferret v7.0 software http://ferret.pmel.noaa.gov/Ferret/.

Cyclone Fani is the strongest pre-monsoon cyclone to form in the Bay of Bengal after 1994 (Fig. 1a). The storm attained cyclonic storm status [wind speed more than 18 m s^−1^ (35 knots)] at 5.2°N on 27th April 1130 a.m. IST. (Fig. 2a). This is the closest formation of a cyclonic storm near to equator in the north Indian Ocean (including both the Arabian Sea and the Bay of Bengal), since 1990. From 29th April 1130 p.m. to 30th April 1130 p.m., Fani intensified rapidly with an increase in wind speed of 20.58 m s^−1^ (40 knots) in a short span of 24 h and it became an extremely severe cyclonic storm with a wind speed of 48.87 m s^−1^ (95 knots). This is the second fastest rapid intensification (in 24 h) among all the pre-monsoon cyclones in the Bay of Bengal during the period 1990–2019 (Fig. 1b). By 2nd May 0230 p.m., it intensified to a peak wind speed of 59.16 m s^−1^ (115 knots) with a well-defined eye. This makes it the strongest pre-monsoon (March–May) Bay of Bengal cyclone after 1994. As per the advanced Dvorak technique (ADT) estimate by Meteosat 8 satellite (MSG1), the maximum temperature recorded in the eye was 17.74 °C on 2nd May 0745 p.m. This indicates a very warm eye as compared to the surroundings. On 3rd May, between 0800 a.m. to 1000 a.m., it hit the Odisha coast near Puri with a wind speed of ~ 54 m s^−1^ (105 knots). Also, it maintained a wind speed of ≥ 51.44 m s^−1^ (≥ 100 knots) for 36 h, which is the most prolonged duration by a pre-monsoon cyclone in the Bay of Bengal, after 1990. As cyclone Fani approached the Odisha coast, the high winds resulted in coastal storm surge and flooding. The wave rider buoy offshore at Gopalpur, Odisha (118 km south of Puri), reported a wave height of about 5.2 m on early morning hours of 3rd May (local time). Another wave rider buoy at Digha, West Bengal, reported a wave height of 4 m during the late night of 3rd May (local time).

During its lifespan, cyclone Fani attained ACE of 16.66 (10^4^ kt^2^). ACE of cyclone Fani was about four times larger than the climatology for the pre-monsoon (March–May) Bay of Bengal cyclones, which is 4.25 (10^4^ kt^2^), during the period 1990–2018. Also, ACE of cyclone Fani is the largest by any pre-monsoon cyclone since 1990 in the north Indian Ocean (including both the Arabian Sea and the Bay of Bengal).

### Factors controlling the genesis and rapid intensification of cyclone Fani

**Figure 3:**
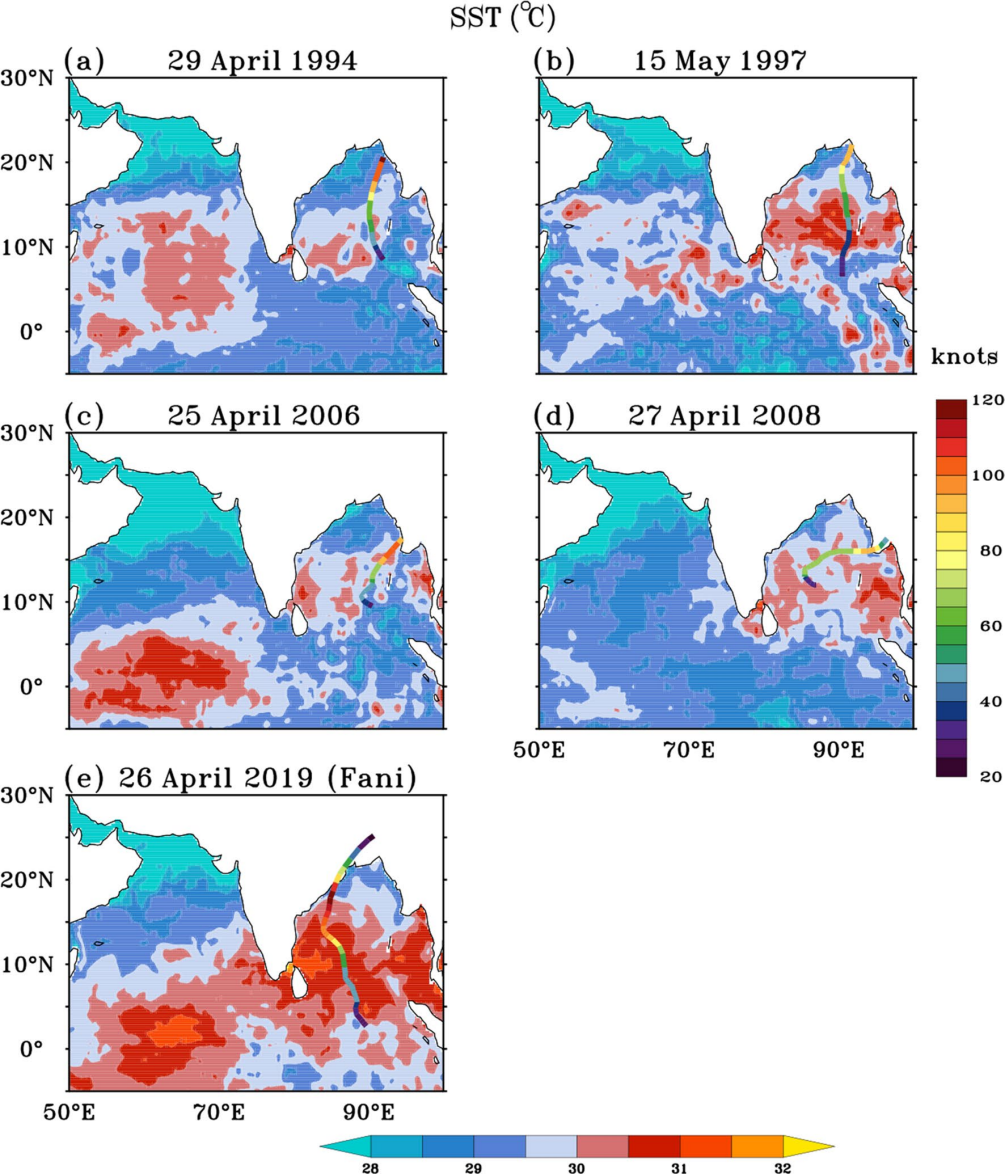
(**a**–**e**) SST (°C, shaded) averaged over 7 days prior to the genesis of five extremely severe cyclone [wind speed ≥ 46.3 m s^−1^ (90 knots)] in the Bay of Bengal during the pre-monsoon season. In each panel cyclone track is overlaid over the SSTs. Colour along the cyclone track denotes the wind speed in knots. Dates above each panel denote the genesis date of each cyclone. The figure is created using Ferret v7.0 software http://ferret.pmel.noaa.gov/Ferret/.

**Figure 4:**
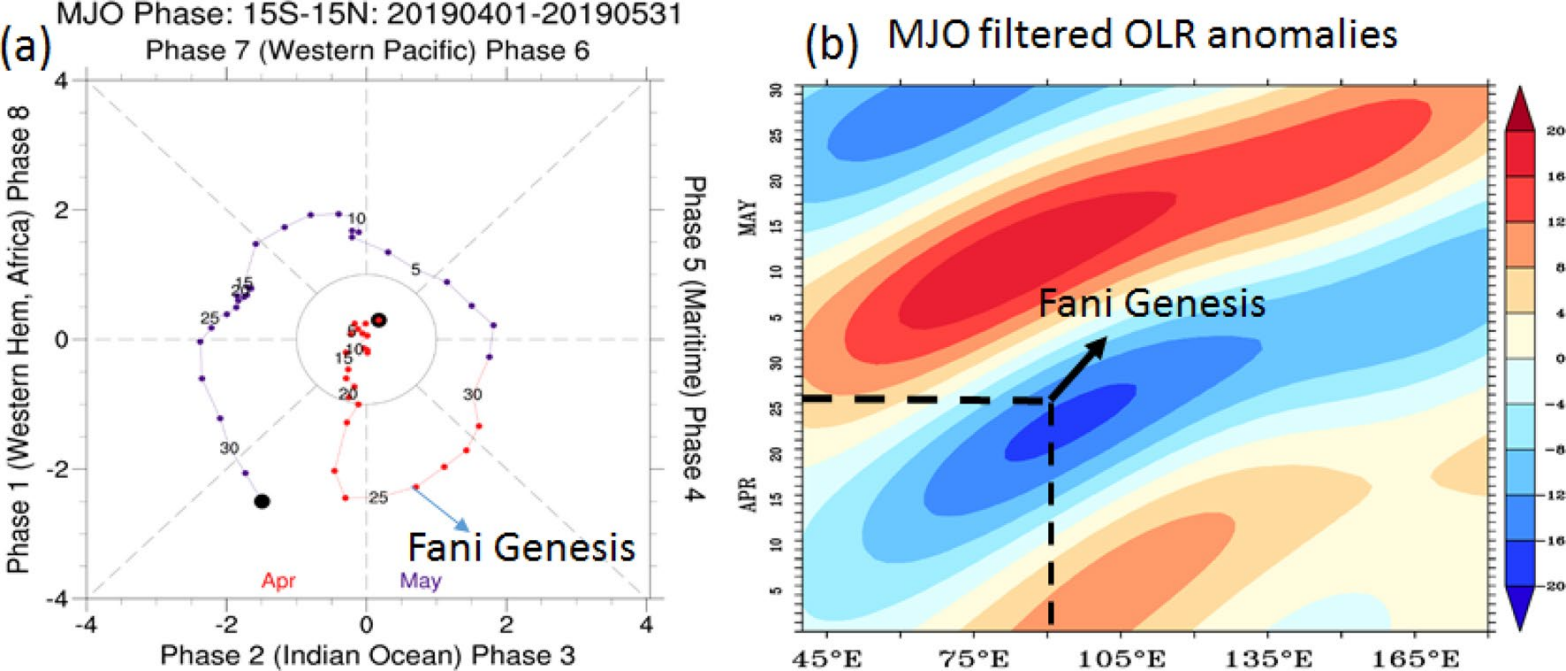
(**a**) Phase diagram of MJO RMM from 1st April 2019 to 31st May 2019. Colour denotes the propagation in April and May. When the index is within the circle in the centre, the MJO activity is considered as weak, and when it is outside the circle it is considered as strong. (**b**) Propagation of space–time filtered OLR anomalies (W m^−2^) from April 2019 to May 2019. Blue shades denote enhanced convection and red shades denote suppressed convection. The figure is created using Ferret v7.0 software http://ferret.pmel.noaa.gov/Ferret/.

**Figure 5:**
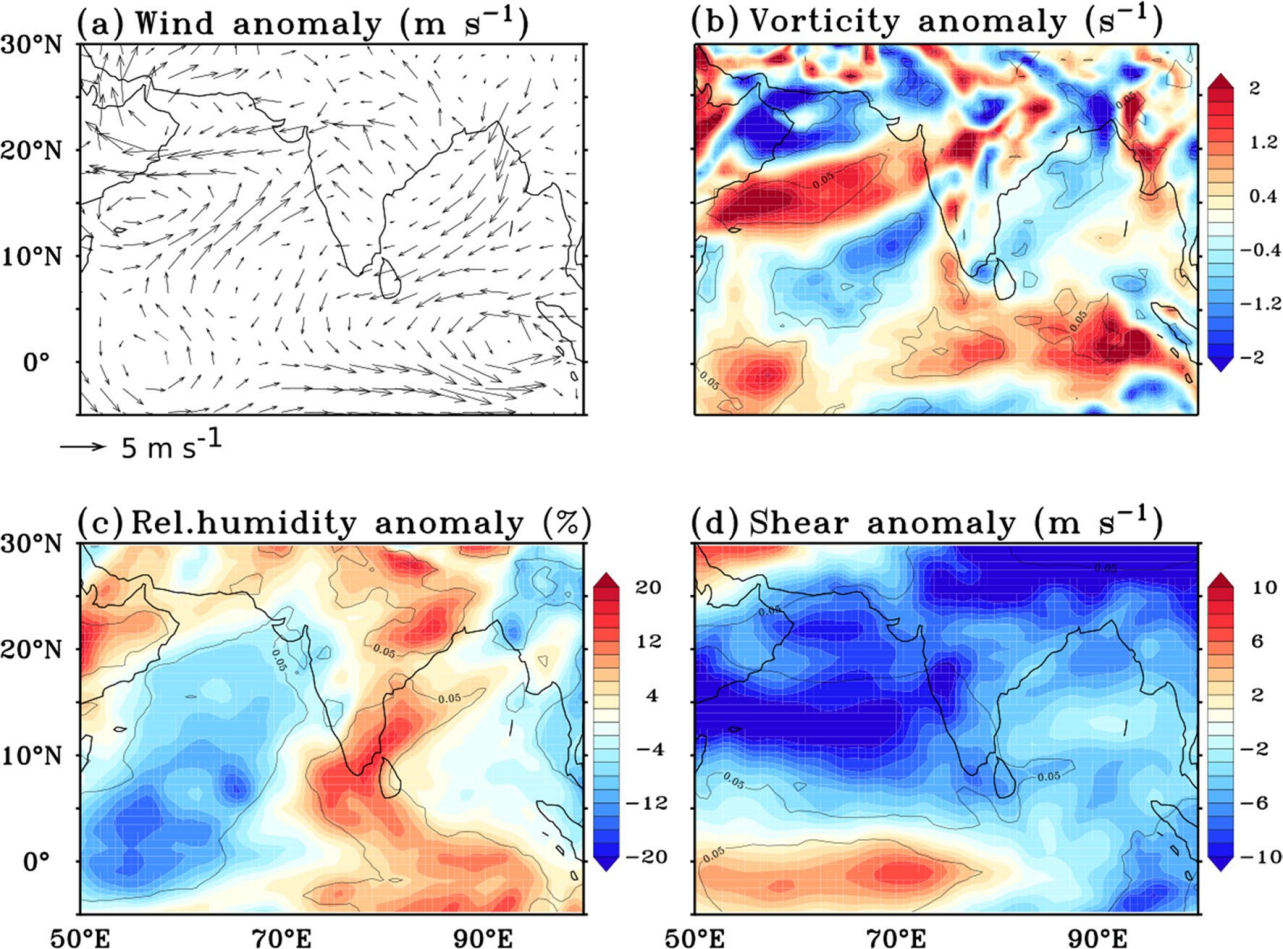
(**a**) 850 hPa Wind (m s^−1^), (**b**) 850 hPa relative vorticity (10^−5^, s^−1^), (**c**) relative humidity (1000–500 hPa average, %) and (**d**) wind shear (m s^−1^) anomalies. Black contour line in the panels denote the 95% confidence level. Anomalies of all the parameters are averaged for the period 19–25 April 2019. The figure is created using Ferret v7.0 software http://ferret.pmel.noaa.gov/Ferret/.

A week prior (19–25 April) to the formation of cyclone Fani, high SSTs of the order of 30–31 °C with significantly (*P* < 0.05) large SST anomalies of about 0.8–1.2 °C were observed over most parts of the Bay of Bengal (Fig. 2a), providing conducive environment for cyclone formation and intensification. These are the highest SSTs observed spatially in the Bay of Bengal prior to the genesis of the last five extremely severe cyclones in the pre-monsoon season (Fig. 3a–e). The SSTs averaged for a week (day -7 to day -1) prior to the day of cyclogenesis (day 0), over a 5° × 5° region at the genesis center, shows that the average SST prior to genesis of cyclone Fani was 30.4 °C. This is the highest SST observed at the genesis center prior to genesis of any cyclone in April month in the Bay of Bengal during the period 1990–2019. Also, from 25th April onwards, an active propagation of the MJO in the eastern Indian Ocean (phase 3) was observed (Fig. 4a). From 28th April, the MJO activity started moving towards the Maritime Continent (phase 4). Enhanced convection, as indicated by negative OLR anomalies, can be seen rapidly propagating towards the Maritime Continent (Fig. 4b). As the MJO propagated from the western to the eastern Indian Ocean and later to the maritime region, it led to anomalous westerlies in the equatorial region in the Bay of Bengal along its trailing edge of enhanced convection. Also, anomalous easterlies were seen in the Bay of Bengal at 10°N–15°N (Fig. 5a). This led to an anomalous cyclonic circulation over the southern Bay of Bengal and increased the low-level cyclonic vorticity (Fig. 5b). This anomalous low-level cyclonic vorticity is a favourable condition for the genesis of the cyclone^11^.

High relative humidity (moist atmosphere) in the low-to-middle troposphere is another crucial factor that provides conducive conditions for the genesis and the intensification of the cyclone^28,29^. The MJO propagation causes changes in the relative humidity that affects the genesis of the cyclone^30^. Generally, anomalously high relative humidity is observed over the area where an active MJO related convection is present. Figure 5c displays the average anomalous relative humidity from 1000 to 500 hPa averaged over the period 19th–25th April. It is seen that significantly (*P* < 0.05) anomalous relative humidity was persisting over the east Equatorial Indian Ocean during this period.

Along with relative humidity, the vertical wind shear of horizontal winds between upper and lower levels plays a significant role in the genesis and intensification of cyclones, where high wind shear inhibits cyclone formation and low wind shear favours it^7,31,32^. One week before cyclone formation, as the MJO enhanced convective moved from the western Indian Ocean to the eastern Indian Ocean, the vertical wind shear over the southern Bay of Bengal has decreased as shown by a negative anomaly (Fig. 5d). Previous studies show that the active propagation of MJO through the Bay of Bengal leads to a decrease in wind shear. This decrease in wind shear is due to anomalous low-level westerlies and anomalous upper-level easterlies, which counteract the climatological prevailing zonal wind in the lower and upper levels over the southern Bay of Bengal^19^. Thus, in the case of Fani, active MJO propagation through the Indian Ocean provided conducive atmospheric conditions for cyclogenesis in the southern Bay of Bengal. These favourable atmospheric conditions, coupled with anomalous high SSTs, led to the genesis of cyclone Fani on 26th April.

**Figure 6:**
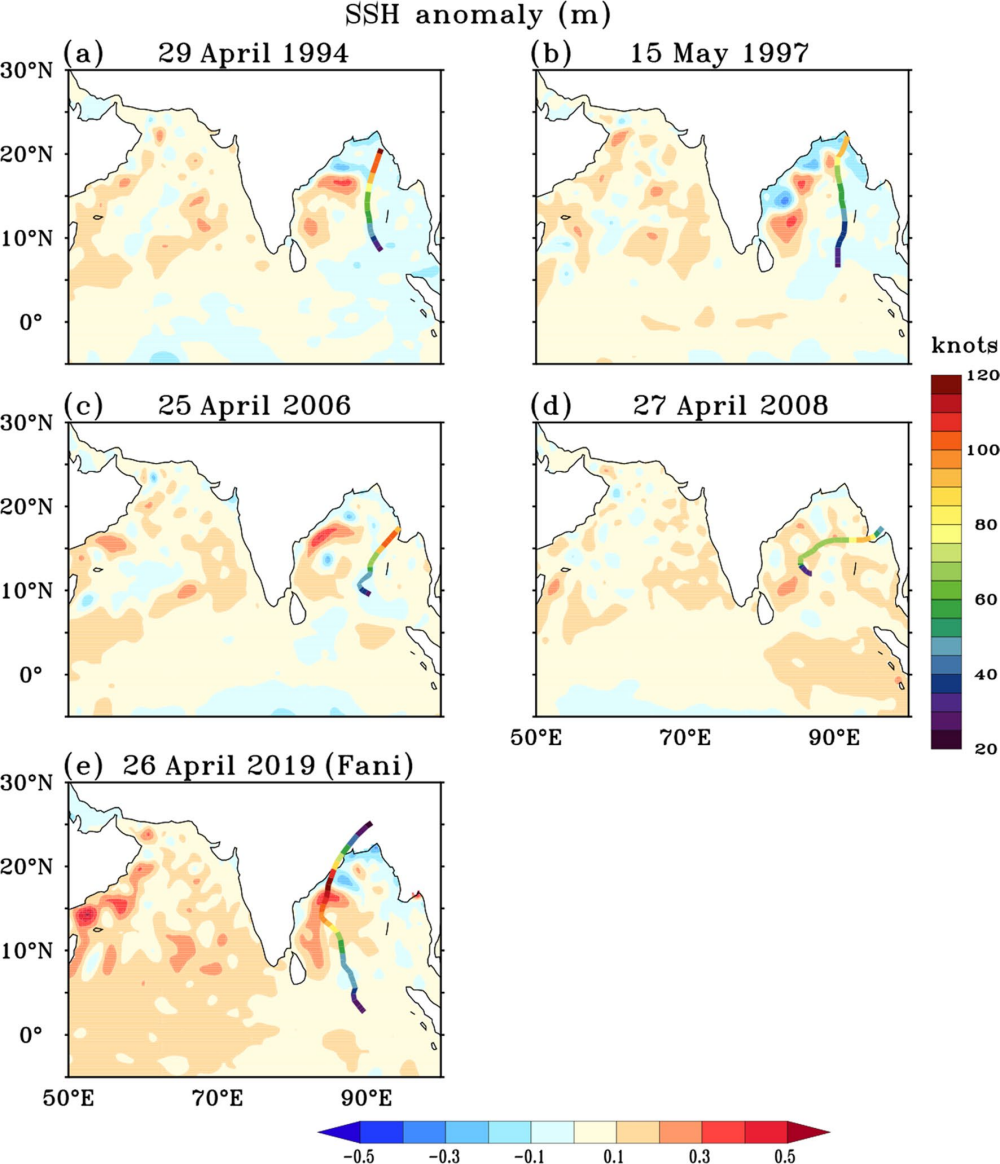
(**a**–**e**) SSH anomalies (m, shaded) averaged over the 7 days prior to the genesis of five extremely severe cyclone [wind speed ≥ 46.3 m s^−1^ (90 knots)] in the Bay of Bengal during the pre-monsoon season. In each panel cyclone track is overlaid over the SSH anomalies. Colour along the cyclone track denotes the wind speed in knots. Dates above each panel denote the genesis date of each cyclone. The figure is created using Ferret v7.0 software http://ferret.pmel.noaa.gov/Ferret/.

After the genesis of cyclone Fani, it remained in a region of high vertical wind shear, larger than 20 knots, from 26th April–27th April leading to slow intensification in its initial stage. From 28th April onwards, Fani moved into a region of low to moderate shear with values in the range of 5.14–7.17 m s^−1^, which was conducive for intensification (figure not shown). Also, the TCHP was very high along the path of cyclone Fani with values in the range 75–100 kJ cm^−2^ (Fig. 2e). From 29th April 1130 p.m.–30th April 1130 p.m., it underwent rapid intensification with an increase in wind speed of 20.58 m s^−1^ (40 knots) and became an extremely severe cyclone with a wind speed of 48.87 m s^−1^ (95 knots) on 30th April 1130 p.m. This rapid intensification occurred as cyclone Fani moved over the area of an anticyclonic warm-core oceanic eddy which was present over the region (11°N–14°N, 85°E–88°E, See Fig. 2c) during the period 19th–25th April (represented by anomalous sea surface height of the order of 20–30 cm). These warm-core oceanic eddies are known to influence the cyclone intensity in different ocean basins. Sudden intensification of Hurricane Opal from 965 to 916 hPa in the Gulf of Mexico over 14 h after passing over a warm core eddy^22^ is an excellent example of the influence of warm-core eddy on cyclones. In the north Indian Ocean also the mesoscale eddies are commonly observed^33^. Theses eddies are generated due to ocean local instability and also they propagate from the eastern or central Bay of Bengal to the western Bay of Bengal^33,34^. In the pre-monsoon season, these eddies in the western Bay of Bengal move northeastward along the east India coastal current^35^. The cyclonic and anticyclonic eddies significantly affect the intensity of the cyclone when the cyclones cross these eddies^1^. The warm-core eddy provides more warm water volume and thus aids intensification of the cyclone as the cyclone move over these eddies^36^. From Fig. 6 it can be seen that cyclone Fani was the only extremely severe cyclone among the five extremely severe cyclones, which has directly passed over the warm core eddy present over the west Bay of Bengal. There was basin wide very warm SSTs of order of 29–31 °C (Fig. 3e) and high TCHP of about 80–100 kJ cm^−2^ up to 15°N (Fig. 2e) along the track of cyclone Fani during the period 19th–25th April. This has aided the cyclone in maintaining a very high wind speed of ≥ 51.44 m s^−1^ (≥ 100 knots) for a record time period of 36 h.

As the MJO further propagated eastwards in the first week of May (Fig. 4a), the relative vorticity, relative humidity and the wind shear averaged over 5° × 5° box at the region where cyclone Fani had its genesis on 26th April, again became unfavourable for cyclogenesis (Supplementary Fig. 1). Further during 4–10 May, i.e. after the landfall of cyclone Fani, SST cooling of 1.5–2 °C was observed along and in the periphery of the cyclone track, with maximum cooling of more than 2 °C in the west-central Bay of Bengal (Fig. 2b). There was a slight reduction in the SSH anomalies (Fig. 2d) and the TCHP also reduced drastically along the track of the cyclone Fani, with maximum decrease observed in the west central Bay of Bengal, where the TCHP declined to less than 20 kJ cm^−2^ (Fig. 2f). This shows that there is a two-way interaction. Large SST warming before the cyclone energizes the cyclone and plays a significant role in its long lifespan and intensification. Thereafter, the strong cyclonic winds enhance the ocean mixing and upwelling of cold water from the sub-surface. Along with persistent cloud cover, this leads to a decrease in SST and TCHP.

## Discussion and summary

Cyclone Fani formed on 26th April 2019 in the Bay of Bengal. It is the strongest pre-monsoon cyclone to form in the Bay of Bengal after 1994. It maintained its intensity as an extremely severe cyclone for 36 h, which is the longest duration by a pre-monsoon Bay of Bengal cyclone since 1990. An active propagation of MJO through the Indian Seas during 19th–25th April led to anomalous westerlies in the near-equatorial region and increased the cyclonic vorticity over the south Bay of Bengal, providing conditions favourable for cyclogenesis. The enhanced vorticity was supported by a decrease in the vertical wind shear, further enhancing the atmospheric conditions for the genesis of the cyclone.

Along with conducive atmospheric conditions, anomalous warm ocean temperatures led to the genesis of the cyclone Fani on 26th April. Earlier studies show that SST plays a significant role in enhancing the accumulated cyclone energy, which is reflected in the long duration and high intensity of cyclones^12,37^. Here we show that favourable ocean subsurface conditions with a warm-core ocean eddy provided a vast reservoir of heat to the cyclone that fuelled it and led to its rapid intensification to an extremely severe cyclone. This shows that along with the SSTs, the ocean subsurface and the presence of ocean eddies also play an essential role in governing the intensity of cyclone.

Pre-monsoon Bay of Bengal cyclones are intensifying in the recent decades due to enhanced large-scale monsoon circulation, which is associated with enhancement of lower-level cyclonic and upper-level anticyclonic anomalies due to local atmospheric warming^3^. Since the Bay of Bengal is landlocked and surrounded by densely populated coastlines, it is necessary to closely monitor the basin for future storms as climate models project continued warming of the Indian Ocean^38^.

## Supplementary Information

Supplementary Figure.

## Data Availability

Cyclone Fani data such as wind speed and track information are obtained from the India Meteorological Department (IMD) Regional Specialised Meteorological Centre (RSMC) preliminary report on Fani cyclone. The data for other cyclones during the period 1990–2018 were obtained from RSMC, IMD website http://www.rsmcnewdelhi.imd.gov.in. Daily SST data (OISST data) is obtained from the NOAA Physical Sciences Laboratory website https://psl.noaa.gov/. The wave height data is obtained from the Indian National Centre for Ocean Information Services (https://incois.gov.in). Daily data for sea level anomalies are obtained from the Copernicus Marine Service Altimeter satellite gridded Sea Level Anomalies L4 dataset (https://resources.marine.copernicus.eu/) for the period 1993–2019.The cyclone eye temperature data is obtained from the advanced Dvorak technique (ADT) estimate by Meteosat 8 satellite (MSG1), (http://tropic.ssec.wisc.edu/). The MJO RMM index data is obtained from the Australian Bureau of Meteorology (http://www.bom.gov.au/climate/mjo). Daily data for relative vorticity (850 hPa), wind (850 hPa and 200 hPa), relative humidity (1000–500 hPa) for the period 1982–2019 is obtained from Era Interim reanalysis dataset website https://apps.ecmwf.int/datasets/data/. The OLR data is obtained from NOAA interpolated OLR dataset https://psl.noaa.gov/.
