# Supplementary material for: Role of warm ocean conditions and the MJO in the genesis and intensification of extremely severe cyclone Fani
Source: Sci Rep. 2021 Feb 11;11:3607. doi: 10.1038/s41598-021-82680-9 (PMC7878906; doi:10.1038/s41598-021-82680-9)
Supplement: Supplementary file 1 — Supplementary Figure. [file 41598_2021_82680_MOESM1_ESM.docx]

**Supplementary information for**

**Role of warm ocean conditions and the MJO in the genesis and intensification of extremely severe cyclone Fani**

Vineet Kumar Singh*, M.K. Roxy, and Medha Deshpande

*Corresponding author: Vineet Kumar Singh ([vineetsingh.jrf@tropmet.res.in](mailto:vineetsingh.jrf@tropmet.res.in))

**Supplementary Figure caption**

**Supplementary Figure 1.** Time series of (a) Relative vorticity (10^-5^, s^-1^) (b) Vertically averaged relative humidity (1000-500 hPa avg., %) (c) Wind shear (m s^-1^) anomalies during the period 19 April 2019 – 10 May 2019 averaged over 5°x5° box around the genesis center. The figure is created using Ferret v7.0 software <http://ferret.pmel.noaa.gov/Ferret/>.


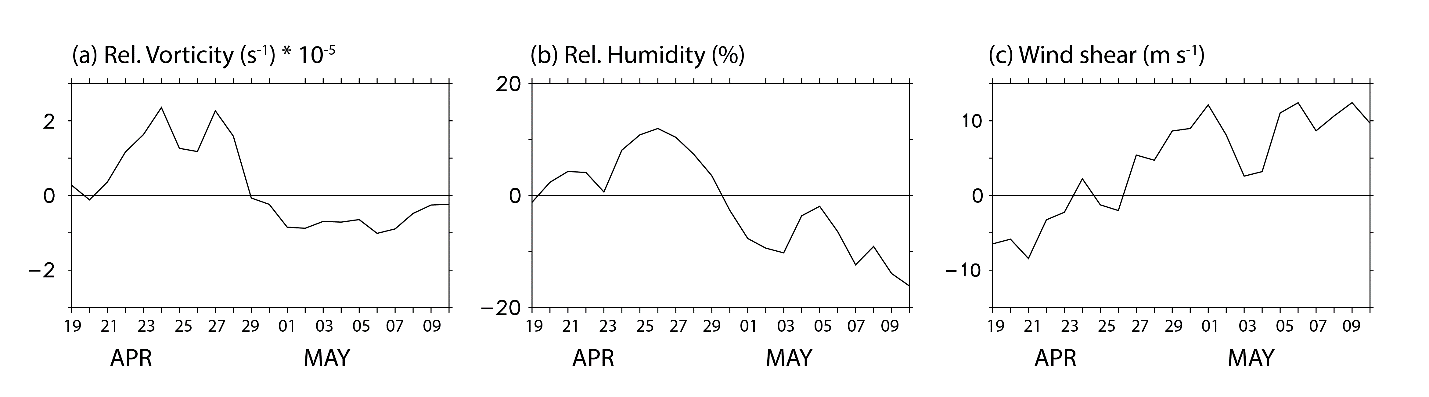


**Supplementary Figure 1**. Time series of (a) Relative vorticity (10^-5^, s^-1^) (b) Vertically averaged relative humidity (1000-500 hPa avg., %) (c) Wind shear (m s^-1^) anomalies during the period 19 April 2019 – 10 May 2019 averaged over 5°x5° box around the genesis center. The figure is created using Ferret v7.0 software <http://ferret.pmel.noaa.gov/Ferret/>.
